# The Intrinsic Neuronal Activation of the CXCR4 Signaling Axis Is Associated with a Pro-Regenerative State in Cervical Primary Sensory Neurons Conditioned by a Sciatic Nerve Lesion

**DOI:** 10.3390/ijms26010193

**Published:** 2024-12-29

**Authors:** Petr Dubový, Ivana Hradilová-Svíženská, Václav Brázda, Anna Jambrichová, Viktorie Svobodová, Marek Joukal

**Affiliations:** 1 Department of Anatomy, Cellular and Molecular Research Group, Faculty of Medicine, Masaryk University, Kamenice 3, CZ-625 00 Brno, Czech Republic; 2 Institute of Biophysics, Czech Academy of Sciences, Královopolská 135, CZ-612 65 Brno, Czech Republic

**Keywords:** sciatic nerve, transection, pre-conditioning, axon regeneration, AMD3100, IL-6, STAT3

## Abstract

CXCL12 and CXCR4 proteins and mRNAs were monitored in the dorsal root ganglia (DRGs) of lumbar (L4–L5) and cervical (C7–C8) spinal segments of naïve rats, rats subjected to sham operation, and those undergoing unilateral complete sciatic nerve transection (CSNT) on post-operation day 7 (POD7). Immunohistochemical, Western blot, and RT-PCR analyses revealed bilaterally increased levels of CXCR4 protein and mRNA in both lumbar and cervical DRG neurons after CSNT. Similarly, CXCL12 protein levels increased, and CXCL12 mRNA was upregulated primarily in lumbar DRGs ipsilateral to the nerve lesion. Intrathecal application of the CXCR4 inhibitor AMD3100 following CSNT reduced CXCL12 and CXCR4 protein levels in cervical DRG neurons, as well as the length of afferent axons regenerated distal to the ulnar nerve crush. Furthermore, treatment with the CXCR4 inhibitor decreased levels of activated Signal Transducer and Activator of Transcription 3 (STAT3), a critical transforming factor in the neuronal regeneration program. Administration of IL-6 increased CXCR4 levels, whereas the JAK2-dependent STAT3 phosphorylation inhibitor (AG490) conversely decreased CXCR4 levels. This indicates a link between the CXCL12/CXCR4 signaling axis and IL-6-induced activation of STAT3 in the sciatic nerve injury-induced pro-regenerative state of cervical DRG neurons. The role of CXCR4 signaling in the axon-promoting state of DRG neurons was confirmed through in vitro cultivation of primary sensory neurons in a medium supplemented with CXCL12, with or without AMD3100. The potential involvement of conditioned cervical DRG neurons in the induction of neuropathic pain is discussed.

## 1. Introduction

The activation of the neuronal regenerative program is a crucial intrinsic factor for successful axon reinnervation following peripheral nerve injury [1,2]. The bodies of primary sensory neurons, located in the dorsal root ganglia (DRGs), provide a valuable model for studying the mechanisms that induce the neuronal regeneration program after axotomy. The DRG neurons send off afferent axons, with peripheral arms extending into the peripheral nerves, and central arms entering the spinal cord through the dorsal roots. It is well known that a peripheral nerve injury triggers the regulation of regeneration-associated genes and proteins, highlighting DRG neurons’ regeneration potential [2,3,4]. The regeneration program of DRG neurons promotes the regeneration of peripheral arms in damaged nerves and supports the regeneration of central arms, even under unfavorable conditions in the spinal cord [5]. We have also demonstrated that unilateral sciatic nerve compression (SNC) or complete sciatic nerve transection (CSNT) increases regeneration-associated proteins not only in lumbar (L4–L5) DRG neurons but also in remote cervical (C6–C8) DRG neurons. These results suggest that the intrinsic neuronal regeneration program in DRG neurons along the spinal neuroaxis may be triggered by molecules like IL-6, which are released from axotomized DRG neurons into the cerebrospinal fluid of the paraspinal subarachnoid space [6,7,8]. The pro-regenerative state induced by a sciatic nerve injury in remote cervical DRG neurons can be a useful in vivo model for testing various molecular signaling pathways involved in the neuronal regeneration program activated by a systemic reaction.

Chemokines are small, secreted proteins initially implicated in the regulation of leukocyte chemotaxis during host defense and pathological immune responses. Most chemokines bind to several chemokine receptors, and conversely, most chemokine receptors recognize multiple chemokines [9,10]. Recently, mounting evidence supports the role of chemokine signaling in both physiological and pathological processes within the nervous system [10,11,12].

CXCR4 is unique among chemokine receptors, having only one known ligand, CXCL12, formerly known as stromal-derived factor 1 [13]. The chemokine CXCL12/CXCR4 signaling axis is involved in a broad spectrum of processes associated with nervous tissue development, degeneration, and regeneration [14]. Published results have described the upregulation of both CXCL12 and CXCR4 in DRGs of rodent experimental models of neuropathic pain resulting from peripheral nerve damage [15,16,17]. However, our observations revealed increased levels of both CXCL12 and CXCR4 proteins bilaterally, not only in lumbar DRGs associated with unilateral sciatic nerve injury but also in the remote cervical DRGs [15].

The present experiments were first designed to demonstrate the upregulation of CXCL12 and CXCR4 synthesis in both lumbar and cervical DRGs after unilateral sciatic nerve injury. In addition, our experimental model of unilateral sciatic nerve injury, which triggers a pro-regeneration state in cervical DRG neurons [8], was used to investigate the potential role of CXCR4 signaling in activating the regeneration program in primary sensory neurons. In this context, the relationship between CXCR4 signaling and STAT3 activation was explored after intrathecal application of a specific antagonist of CXCR4 (AMD3110), as well as IL-6 or AG490 as activator or inhibitor of JAK2-dependent phosphorylation of STAT3, respectively. The positive effect of CXCR4 signaling, through its ligand CXCL12, on the axon regeneration capacity of DRG neurons was confirmed in vitro.

## 2. Results

### 2.1. Immunofluorescence Staining and RT-PCR Analysis of CXCL12 and CXCR4 Proteins and mRNAs in DRGs Following Sciatic Nerve Lesion

In our previously published paper [15], we reported increased levels of CXCL12 and CXCR4 proteins in the rat cervical and lumbar DRGs at postoperative day 3 (POD3) and 14 (POD14) after sciatic nerve compression (SNC). To verify the effect of different sciatic nerve lesions on the cellular localization and synthesis of CXCL12 and CXCR4 at POD7, we examined DRGs of cervical (C7–C8) and lumbar (L4–L5) segments harvested from naïve rats and those subjected to sham operation and complete sciatic nerve transection (CSNT). These tissue samples were used for subsequent immunofluorescence staining and RT-PCR analysis.

#### 2.1.1. CXCL12 Immunostaining

**Figure 1:**
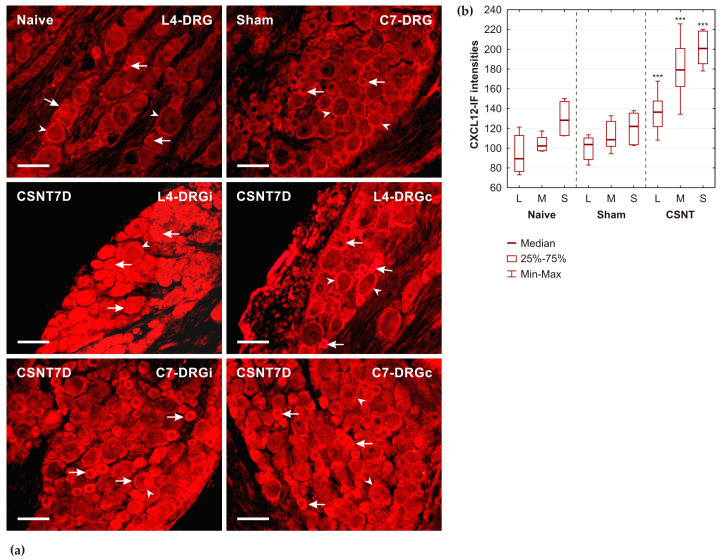
(**a**) Representative sections of DRGs from naïve rats (Naive) and rats undergoing sterile sham (Sham) and CSNT operations on POD7 (CSNT7D). DRGs from CSNT-operated rats were taken from lumbar (L4-DRG) and cervical (C7-DRG) segments on both ipsilateral (i) and contralateral (c) sides. All sections were immunostained for CXCL12 detection under identical conditions. Increased intensities of CXCL12 immunofluorescence were observed in the neuronal bodies (arrows) and satellite glial cells (arrowheads) of both lumbar and cervical DRGs compared with those from naïve or sham-operated rats. Scale bars = 40 µm. (**b**) The results of CXCL12-IF intensities measured in the neuronal cytoplasm of large (L), medium (M), and small (S) cervical DRG neurons from naïve rats (Naïve), as well as both ipsilateral and contralateral DRGs of Sham- and CSNT-operated rats for POD7. *** Significant differences (*p* < 0.001) compared with sham-operated rats, as was determined using a Mann–Whitney U-test.

Sections of the cervical and lumbar DRGs from naïve and sham-operated rats revealed moderate intensity of CXCL12 immunofluorescence (-IF) in some small- and medium-sized neurons, as well as satellite glial cells (SGCs) surrounding mainly large-sized neurons. CSNT resulted in a significant increase in CXCL12-IF in lumbar DRGs ipsilateral to the lesioned nerve, with this increase particularly prominent in neurons of all sizes. Additionally, a significant increase in intensity was observed in SGCs and small- to medium-sized neurons in the contralateral lumbar DRGs. CSNT also led to an increase in CXCL12-IF in cervical DRGs of both sides, with the most pronounced intensities observed in small- and medium-sized neurons and a less noticeable difference in SGCs when compared with DRGs from naïve rats or sham controls (Figure 1a).

The results of the semiquantitative analysis of CXCL12-IF, measured in the neuronal cytoplasm of cervical DRGs from naïve rats, as well as both ipsilateral and contralateral DRGs of sham- and CSNT-operated rats for POD7, are illustrated in Figure 1b.

#### 2.1.2. CXCR4 Immunostaining

**Figure 2:**
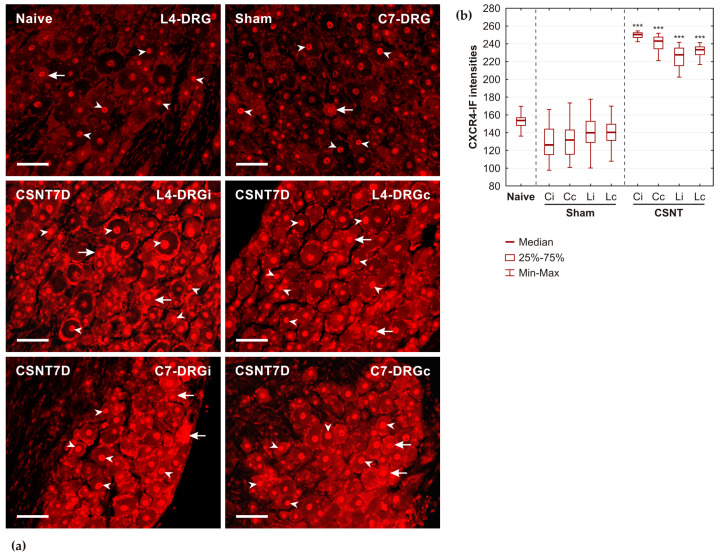
(**a**) Representative sections of DRGs from naïve rat (Naive) and rats after sterile sham (Sham) and CSNT operations on POD7 (CSNT7D). DRGs from CSNT-operated rats were taken from lumbar (L4-DRG) and cervical (C7-DRG) segments on both ipsilateral (i) and contralateral (c) sides. All sections were immunostained for CXCR4 detection under identical conditions. Sciatic nerve lesions induced an increased intensity of CXCR4 immunofluorescence in neuronal nuclei (arrowheads) and a diffuse pattern in the bodies of some DRG neurons (arrows). The heightened intensities of CXCR4 immunofluorescence predominantly loaded nuclei of both DRG neurons and their satellite glial cells. Scale bars = 40 µm. (**b**) The results of CXCR4-IF intensities measured in the neuronal nuclei of the cervical (C) and lumbar (L) DRGs from both ipsilateral (i) and contralateral (c) sides removed from naïve rats (Naïve) as well as from Sham- and CSNT-operated rats on POD7. *** Significant differences (*p* < 0.001) compared with sham-operated rats, as was determined using a Mann–Whitney U-test.

Individual small and medium-sized neuronal bodies in the cervical and lumbar DRGs of naïve and sham-operated rats exhibited diffuse, low intensity of CXCR4-IF. In contrast, the neuronal nuclei displayed a stronger intensity of CXCR4-IF. A similar low intensity of CXCR4-IF was observed in the SGCs and their nuclei. Following CSNT, there was a marked increase in the diffuse CXCR4-IF within some neuronal bodies and a significant enhancement in nuclear intensities in all neurons of lumbar DRGs on both sides. Nuclear localization of CXCR4-IF in neurons was sharply delineated from the diffuse immunofluorescence in the neuronal cytoplasm. A significant increase in intensity was also detected in SGCs. Comparable increases in CXCR4-IF were also found in cervical DRGs of the same animals (Figure 2a).

The results of the semiquantitative analysis of CXCR4-IF intensities in the neuronal nuclei of the cervical and lumbar DRGs from naïve rats, as well as both ipsilateral and contralateral DRGs of sham- and CSNT-operated rats, are illustrated in Figure 2b.

#### 2.1.3. RT-PCR Analysis of CXCL12 and CXCR4 mRNAs

**Figure 3:**
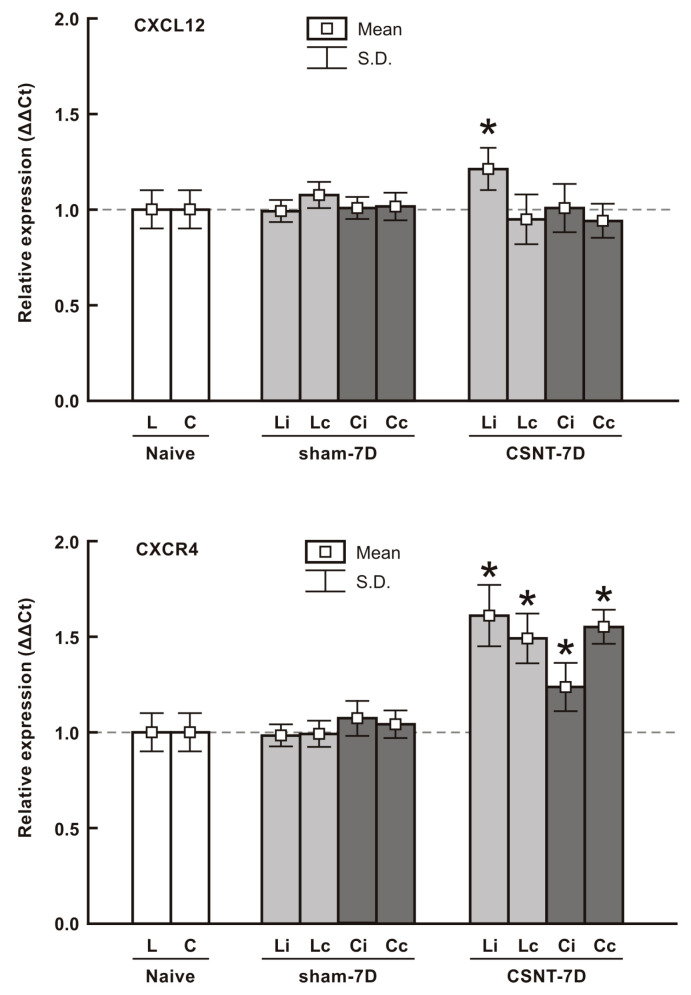
Results of real-time PCR (RT-PCR) of relative CXCL12 and CXCR4 mRNA levels in DRGs of lumbar (L) and the cervical (C) spinal segments (L4–L5) and (C7–C8), respectively, from the ipsilateral (i) and contralateral (c) sides. Tissue samples were collected from naïve rats as well as from sham- and CSNT-operated rats at POD7 (n = 9 for each group). Relative expressions were calculated using Actin as the housekeeping gene and normalized to naïve controls. * Significant differences (*p* < 0.05) compared with sham-operated rats were determined using a Mann–Whitney U-test.

RT-PCR analysis of CXCL12 and CXCR4 mRNAs was performed to assess their levels in the cervical and lumbar DRGs after unilateral CSNT on POD7. These levels were compared with those in DRGs of naïve and sham-operated rats. In sham-operated animals, both cervical and lumbar DRGs showed no significant changes in CXCL12 and CXCR4 mRNA levels compared with naïve controls. A significant increase in CXCL12 mRNA was observed only in lumbar DRGs ipsilateral to CSNT, with no significant changes detected in the contralateral lumbar DRGs or in cervical DRGs on either side. In contrast, CXCR4 mRNA levels were significantly elevated in both cervical and lumbar DRGs on both sides after unilateral CSNT, compared with naïve or sham-operated controls (Figure 3).

#### 2.1.4. Effect of Intrathecal Administration of AMD3100 on CXCL12 and CXCR4 Levels in DRG Neurons and on Axon Regeneration in the Crushed Ulnar Nerve Subsequent to a Prior Sciatic Nerve Lesion

**Figure 4:**
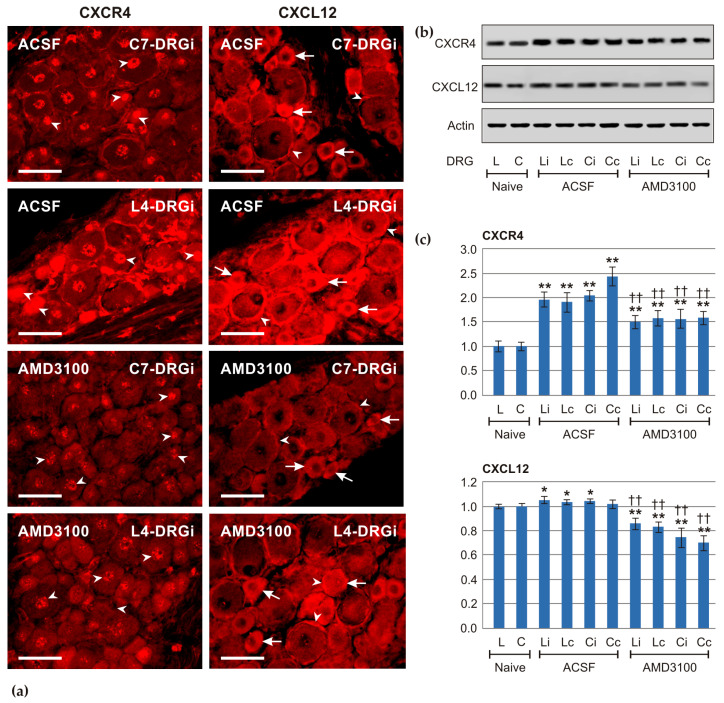
(**a**) The representative sections through the cervical (C7-DRG) and lumbar (L4-DRG) DRGs harvested from the ipsilateral (i) side of rats subjected to CSNT on POD7, and after intrathecal application of artificial cerebrospinal fluid (ACSF) or AMD3100. Sections were incubated under the same conditions to demonstrate CXCR4 and CXCL12 immunofluorescence. The results showed reduced intensities of both CXCL12 and CXCR4 immunofluorescence in DRG sections from rats treated with AMD3100 compared with those treated with ACSF. Changes in CXCR4 immunofluorescence intensities were observed in neuronal nuclei (arrowheads), while changes in CXCL12 immunofluorescence intensities were noted in the neuronal bodies (arrows) and satellite glial cells (arrowheads). Scale bars = 50 µm. (**b**) Representative blots of CXCR4 and CXCL12 proteins with equal protein loading confirmed by actin levels (Actin). Samples of DRGs from both lumbar (L) and cervical (C) segments were collected from naïve rats and from the ipsilateral (i) and contralateral (c) sides of rats undergoing CSNT on POD7, followed by intrathecal application of either artificial cerebrospinal fluid (ACSF) or AMD3100. (**c**) The densitometry results of CXCR4 and CXCL12 protein bands, normalized to Actin levels. Densities of CXCR4 and CXCL12 bands from the naïve DRGs were set as 1 for reference. *, ** denote significant differences (*p* < 0.05, *p* < 0.01, respectively) compared with naïve controls, while †† indicates a significant difference (*p* < 0.01) compared with ACSF-treated counterparts, as determined by the Mann–Whitney U-test.

Immunofluorescence staining and Western blot analysis showed that intrathecal administration of AMD3100 resulted in the expected reduction in CXCR4 protein levels in neurons of both cervical and lumbar DRGs on POD7 compared with rats treated with ACSF. Surprisingly, AMD3100 administration also led to a decrease in CXCL12 protein levels (Figure 4).

**Figure 5:**
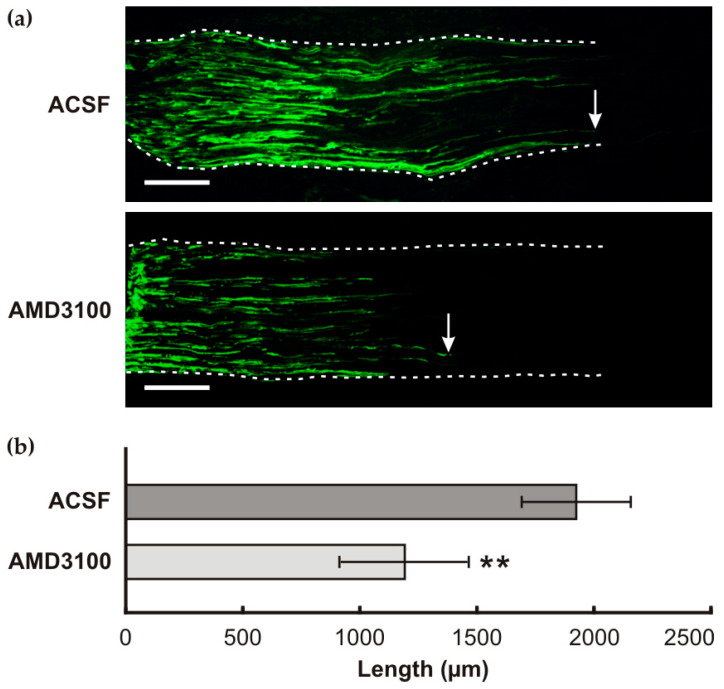
In vivo assay of the pro-regenerative state of cervical DRG neurons conditioned by unilateral CSNT on POD7. The results revealed that intrathecal application of AMD3100 significantly reduced the lengths of SCG10-immunopositive sensory axons regenerated distal to the crush point compared with rats subjected to CSNT and treated with ACSF. (**a**) Representative longitudinal sections through the ulnar nerve distal to the crush site following prior CSNT and intrathecal application of either ACSF or AMD3100. Arrows indicate the maximal length of regenerated axons. Scale bars = 250 µm. (**b**) The lengths of SCG10-immunopositive axons measured distal to the ulnar nerve crush (n = 4 for each group). ** indicates a significant difference (*p* < 0.01) compared with rats treated with ACSF, as determined by the Mann–Whitney U-test.

To investigate the in vivo relationship between CXCR4 signaling and the pro-regenerative state of cervical DRG neurons triggered by a prior sciatic never lesion, we compared the lengths of SCG10-immunostained axons regenerated distal to the UN crush in rats following intrathecal administration of AMD3100 and ACSF. Administration of AMD3100 to rats with CSNT resulted in a significant reduction in the lengths of axons regenerating distal to the UN crush compared with the control group of animals treated with ACSF (Figure 5). The results of our in vivo experimental model using the AMD3100 inhibitor suggested that CXCR4 signaling plays a role in triggering the pro-regenerative neuronal program in cervical DRGs.

#### 2.1.5. In Vitro Effect of CXCR4 Activation on Neurite Outgrowth of Cervical DRG Neurons

To validate the results from the in vivo model regarding the axon regeneration capacity of DRG neurons associated with the CXCR4 signaling axis, primary cultures of cervical DRG neurons were treated with either CXCL12 alone or with CXCL12 supplemented with AMD3100.

**Figure 6:**
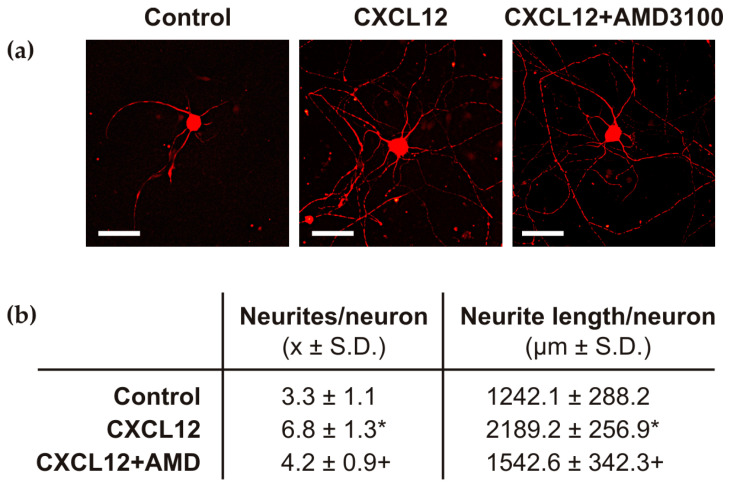
The primary sensory neurons isolated from cervical DRGs were cultivated in vitro (**a**) in control medium (Control), in medium with the addition of CXCL12 (CXCL12), and in medium supplemented with both CXCL12 and AMD3100 (CXCL12+AMD3100). Scale bars = 75 µm. (**b**) The measurement results showed that the addition of CXCL12 into the medium significantly increased the number and length of neurites per neuron. Conversely, the medium supplemented with CXCL12 and AMD3100 reduced the outgrowth of neurites. * denotes a significant difference (*p* < 0.05) compared with DRG neurons cultivated in control medium; † indicates a significant difference (*p* < 0.05) between DRG neurons treated with CXCL12 and CXCL12 + AMD3100 media.

The mean number of neurites per neuronal body increased significantly, and the total length of neurites was significantly higher in the presence of CXCL12-containing medium compared with the control medium. Conversely, the number of neurites sent off by cultured cervical DRG neurons was significantly decreased, and the total length of neurites was significantly reduced when the CXCL12 medium was supplemented with AMD3100 (Figure 6).

#### 2.1.6. Interrelationships Between the CXCR4 Signaling Axis and STAT3 Activation in Cervical DRG Neurons Conditioned by a Sciatic Nerve Lesion

**Figure 7:**
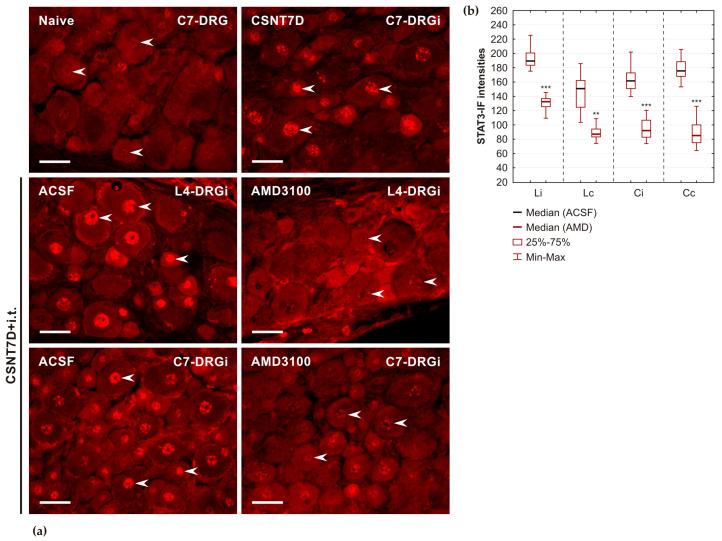
(**a**) The results of STAT3(Y705) immunofluorescence staining are present in representative sections of DRGs. Sections through the cervical (C7-DRG) harvested from a naïve rat (Naïve) and a rat subjected to CSNT on POD7 (CSNT7D). In addition, sections of the cervical (C7-DRGi) and lumbar (L4-DRGi) ganglia ipsilateral (i) to CSNT on POD7 were obtained from rats after intrathecal application of artificial cerebrospinal fluid (ACSF) or AMD3100. The sections were immunostained under identical conditions to detect STAT3(Y705) in the neuronal nuclei (arrowheads). Scale bars = 25 µm. (**b**) Intensities of STAT3(Y705) immunofluorescence measured in the nuclei of DRG neurons (n = 3 for each group) were significantly decreased in DRGs of L4–L5 and C7–C8 segments (L4–L5 DRG, C7–C8 DRG) from both ipsilateral (i) and contralateral (c) sides of rats undergoing CSNT followed intrathecal application of AMD3100, compared with those from ACSF-treated controls. **, *** indicate significant differences (*p* < 0.01 or *p* < 0.001, respectively) compared with DRGs of ACSF-treated rats as determined by the Mann–Whitney U-test.

Bilateral activation of STAT3 and its nuclear translocation were previously demonstrated in lumbar and cervical DRG neurons after unilateral CSNT [18]. In the current experiment, intrathecal administration of AMD3100, an inhibitor of CXCR4, significantly reduced the intensities of nuclear STAT3 in both lumbar and cervical DRGs of rats subjected to unilateral CSNT compared with those of rats receiving ACSF (Figure 7).

**Figure 8:**
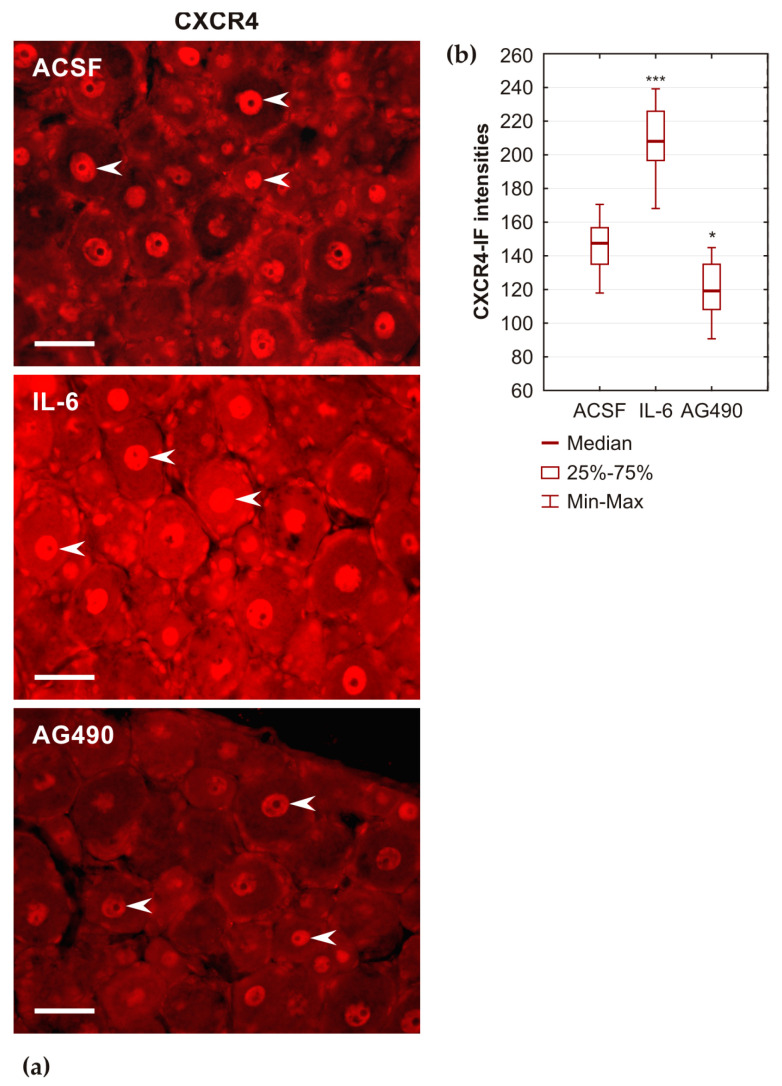
(**a**) The representative sections of cervical DRGs (C7) harvested from rats subjected to CSNT on POD7 followed by intrathecal application of ACSF, IL-6 or JAK2 inhibitor AG490. Sections were immunostained for CXCR4 under identical conditions. Scale bars = 25 µm. (**b**) Measurement of immunofluorescence intensities in neuronal nuclei demonstrated that intrathecal administration of IL-6 increased, while JAK2 inhibitor significantly decreased, the intensities of CXCR4-IF compared to those of control rats treated with ACSF (n = 3 for each group). ***, * denote significant differences (*p* < 0.001 or *p* < 0.05, respectively) compared with DRGs of ACSF-treated rats, as determined by the Mann–Whitney U-test.

The pattern of CXCR4-IF staining in cervical DRGs of rats subjected to CSNT and intrathecal administration of ACSF was comparable to that observed in DRGs removed from rats subjected to CSNT alone. After intrathecal administration of IL-6, cervical DRG neurons showed increased intensities of diffused CXCR4-IF in cytoplasm, and particularly in their nuclei, compared with those treated with ACSF. In contrast, administration of AG490, an inhibitor of JAK2-dependent activation of STAT3, resulted in significantly reduced intensities of CXCR4-IF compared with those treated with ACSF (Figure 8).

#### 2.1.7. Distribution of CXCL12- and CXCR4-Immunostaining Distal to Ulnar Nerve Crush

**Figure 9:**
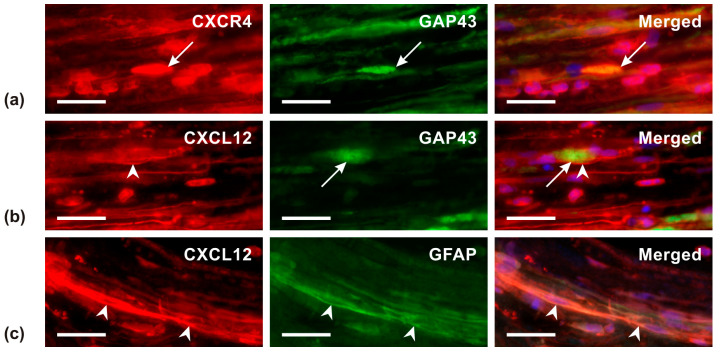
The representative longitudinal sections distal to the UN crush from rats with a prior sciatic nerve lesion for 7 days. The sections were double-immunostained for CXCR4 and GAP43 (**a**) as well as CXCL12 and GAP43 (**b**) or GFAP (**c**). Merged pictures detected immunopositivity for CXCR4 in the growth cones (long arrows) as well as CXCL12 in non-neuronal cells that displayed GFAP immunopositivity indicating their Schwann cell origin (arrowheads). Scale bars = 30 µm.

Double-immunostaining of longitudinal sections revealed the colocalization of CXCR4-IF and GAP43-IF, demonstrating the presence of CXCR4 in growth cones (Figure 9a). Strong intensity of CXCL12-IF was localized in cells and their cytoplasmic processes near GAP43-immunopositive growth cones (Figure 9b). Additionally, double-immunostaining for CXCL12 and GFAP showed that CXCL12-IF was colocalized with Schwann cells (Figure 9c).

## 3. Discussion

The chemokine CXCL12 and its receptor CXCR4 has been detected in neurons and glial cells of both the CNS and PNS [15,16,19]. Utilizing an experimental model of neuropathic pain based on sterile unilateral SNC, our previous results revealed bilaterally increased levels of both CXCL12 and CXCR4 proteins in DRGs. This elevation was observed not only in lumbar DRGs associated with the injured nerve but also in cervical DRGs, which are not connected to the affected nerve. The results of behavioral tests in the same rats demonstrated that changes in CXCL12 and CXCR4 protein levels occurring only in the ipsilateral lumbar DRGs were specifically associated with nerve injury-induced neuropathic pain. However, the alterations in CXCL12 and CXCR4 levels in cervical DRGs on both sides remain unclear [15].

In our current experiments, we confirmed and expanded our understanding that unilateral CSNT also induced a significant increase in both mRNA and protein levels of CXCL12 and CXCR4 in the cervical and lumbar DRGs. The POD7 in our experiments was chosen based on the peak occurrence of cellular and molecular changes associated with Wallerian degeneration distal to the nerve injury [20,21]. Tissue array sections, processed under identical conditions, revealed a significant increase in the intensities of CXCL12-IF in neuronal bodies and SGCs of both the cervical and lumbar DRGs after CSNT compared with DRGs of naïve and sham-operated controls. In addition to the cytoplasmic localization, the current immunodetection revealed a distinct nuclear immunoreactivity of CXCR4 in DRG neurons and, at a lower level, in their SGCs.

The question arises: What is the function of elevated CXCR4 in DRGs non-associated with the damaged nerve? In previous experiments, we observed a significant increase in IL-6 protein and mRNA in cervical DRGs induced by unilateral sciatic nerve injury [6,22]. The increase in IL-6 protein was correlated with the activation and nuclear translocation of STAT3, associated with elevated levels of pro-regenerative protein markers GAP43 and SCG10, as well as a more extensive axon regeneration distal to the UN crush [8,18]. Therefore, in the present experiments, we investigated whether the sciatic nerve injury-induced elevation of CXCR4 in the primary sensory neurons of remote DRGs can initiate a neuronal regenerative program, like IL-6.

### 3.1. CXCR4 Signaling and the Pro-Regenerative State in Cervical DRG Neurons Conditioned by a Sciatic Nerve Lesion

Intrathecal administration of the CXCR4 inhibitor AMD3100 reduced protein levels of CXCR4 in lumbar and cervical DRG neurons on both sides after CSNT. This effect of AMD3100, a non-peptide-specific inhibitor of CXCR4 [23,24], was demonstrated by the results of semiquantitative measurement of CXCR4-IF intensities in neuronal nuclei and confirmed by Western blot analysis. Moreover, the results of both immunofluorescence detection and Western blot analysis after AMD3100 treatment support the specificity of the antibody used to detect CXCR4. In addition to reduction in CXCR4, intrathecal administration of AMD3100 following CSNT surprisingly decreased protein levels of CXCL12 in both the cervical and lumbar DRGs. These findings are consistent with published results demonstrating a significant reduction in both CXCR4 and CXCL12 protein levels following AMD3100 treatment [25,26]. However, the exact mechanism of this AMD3100 effect on the level of CXCL12 protein in DRGs is not known.

A reduction in CXCR4 levels in cervical DRG neurons after AMD3100 administration was also observed in rats, which showed a significant decrease in the length of regenerated afferent axons distal to the UN crush compared with the control group. These results indicate that CXCR4 signaling is involved in the pro-regenerative neuronal program and the axon-promoting state of cervical DRG neurons conditioned by CSNT. The involvement of CXCR4 signaling in the axon-promoting state of DRG neurons was supported by an in vitro assay. The medium supplemented with CXCL12 significantly increased neurite outgrowth of DRG neurons, while the neurite-promoting effect of CXCL12 was reduced by the addition of the CXCR4 inhibitor AMD3100. The promotion of axon regeneration induced by CXCR4 signaling distal to the UN crush is consistent with published results. These studies have demonstrated that intrathecal application of CXCL12 following rat spinal cord injury enhances the sprouting of corticospinal tract axons into the non-permissive environments of white and grey matter. Additionally, CXCL12 has been shown to reduce the repulsion of axonal growth cones of DRG neurons towards myelin [27]. Furthermore, CXCL12/CXCR4 signaling has been observed to stimulate neurite outgrowth in mature retinal ganglion cells (RCGs) cultured in vitro [28,29].

### 3.2. Interrelationships Between the CXCR4 Signaling Axis and Activated STAT3 in Cervical DRG Neurons Conditioned by a Sciatic Nerve Lesion

The activation of STAT3 by JAK2-dependent phosphorylation at the tyrosine-705 (Y705) and subsequent nuclear translocation is linked with the upregulation of regeneration-associated genes and proteins [3,30,31,32,33]. In our previous experiments, we observed that unilateral sciatic nerve lesion induced activation and nuclear translocation of STAT3 bilaterally, not only in lumbar but also in cervical DRG neurons. This effect of a conditioning sciatic nerve lesion is likely mediated by IL-6 released into the CSF of the paraspinal subarachnoid space [6,7,8,18]. The phenomenon of G protein-independent CXCR4 signaling was described in the context of CXCL12-induced transient association of JAK2 with CXCR4, which subsequently led to the activation and nuclear translocation of STAT3 protein [34].

To investigate potential interactions between CXCR4 signaling and STAT3 in the pro-regeneration state of cervical DRG neurons induced by unilateral sciatic nerve lesions, we administered AMD3100 intrathecally and analyzed the activation and nuclear translocation of STAT3. Semiquantitative measurement of immunofluorescence intensities demonstrated that intrathecal application of CXCR4 signaling inhibitor significantly reduced activation and nuclear translocation of STAT3 in both lumbar and cervical DRG neurons after a sciatic nerve lesion. On the other hand, intrathecal administration of IL-6 significantly increased, while the JAK2 inhibitor AG490 decreased immunofluorescence intensities of CXCR4 in DRG neurons compared with those of control animals treated with ACSF. These results suggest a close interrelationship between the CXCL12/CXCR4 signaling axis and IL-6-induced activation of STAT3 in cervical DRG neurons conditioned by sciatic nerve lesions. Furthermore, activated STAT3 is a key transforming factor in initiating the neuronal regeneration program [34]. Therefore, our findings support the involvement of CXCL12/CXCR4 signaling in triggering the pro-regenerative state of DRG neurons.

Some studies have demonstrated the nuclear localization of activated STAT3 and CXCR4, as well as their mutual interactions associated with malignancy [35,36,37,38]. CXCR4 is a highly conserved seven-transmembrane-spanning G protein-coupled receptor which triggers different downstream signaling pathways, including PI3K/AKT/mTOR for modulation of cell proliferation, JAK2/STAT3 for gene transcription, and Ras/MAPK for chemotaxis to promote cell migration [39]. It has been demonstrated that CXCR4 signaling can facilitate a regeneration program in adult RGCs [28,29]. However, while CNTF induced STAT3 phosphorylation in the primary culture of adult RGCs, no STAT3 activation was detected in CXCL12-treated RGCs. This suggests that the JAK2/STAT3 pathway is not activated in RGCs by CXCL12, even though the CXCR4 effect was abrogated by a specific CXCR4 antagonist. Additionally, the axon growth-promoting effect of CXCL12 in RGCs was diminished by inhibiting the PI3K/AKT/mTOR pathway, indicating a likely signaling pathway [29]. This was consistent with the continuous decrease in mTOR-dependent phosphorylation of S6 over extended time after axotomy, suggesting that CXCL12 can modulate mTOR activity via PI3K/AKT signaling [40]. Our results, however, clearly demonstrated a reduction in the activation and nuclear translocation of STAT3 in DRG neurons following the inhibition of CXCR4 signaling. Conversely, there was an increase in nuclear CXCR4 after the activation of STAT3 by IL-6 and its reduction by the action of AG490 inhibitor. This provides evidence that the activation of CXCR4 in primary sensory neurons, whether directly or indirectly associated with axotomy, is linked to the JAK2/STAT3 signaling pathway. The difference in STAT3 activation by CXCR4 signaling between DRG neurons presented here and RGCs [29] may reflect differences in the activation of the regeneration program in neurons of peripheral and central origin.

### 3.3. Local Involvement of CXCR4 Signaling in the Outgrowth of Afferent Axons Distal to a Nerve Injury

The axon-promoting effect of the CXCL12/CXCR4 signaling axis has been demonstrated in motor axons through both in vivo and in vitro experiments. Cellular distribution analysis showed that CXCR4 was present in growing motor axons, while its ligand, CXCL12, was detected in mesenchymal cells surrounding the ventral side of the neural tube and in Schwann cells distal to nerve injury [41,42,43]. Consequently, it is crucial to consider the local interaction between CXCL12 and CXCR4 in the axon-promoting effect in our in vivo test using the UN crush model. Distal to the UN crush, CXCR4-IF was found in growth cones of regenerating axons, whereas CXCL12-IF was detected in activated Schwann cells. Thus, a local effect of CXCR4 and CXCL12 interaction in the regenerated afferent axons and their Schwann cells cannot be excluded distal to the UN crush. However, we do not know whether intrathecal administration of AMD3100 affects CXCR4 distal to the UN crush. Nevertheless, the demonstrated relationship between CXCR4 signaling and the activation and nuclear translocation of STAT3 in DRG neurons in our experiments highlights the significance of CXCR4 signaling for the activation of the neuronal regeneration program.

### 3.4. A Possible Further Involvement of the CXCCL12/CXCR4 Signaling Axis in Cervical DRG Neurons Conditioned by a Sciatic Nerve Lesion

A growing body of experimental evidence describes the potential role of CXCL12/CXCR4 signaling in DRGs and other structures of somatosensory pathways as a factor related to the induction of neuropathic pain [16,17,44,45,46,47]. The development of neuropathic pain involves a complex interplay of cellular and molecular pathophysiological changes in both peripheral tissues and the central nervous system [48,49]. Increased levels of CXCL12 and CXCR4 in ipsilateral lumbar DRGs, where primary sensory neurons are anatomically connected to the injured sciatic nerve, significantly contribute to neuronal hyperexcitability and enhanced regeneration capacity associated with neuropathic pain [16,50]. In line with this, we found that the upregulation of CXCL12 and its receptor CXCR4 in lumbar DRGs ipsilateral to the sciatic nerve lesion is specifically associated with hyperalgesia in the glabrous skin of rat hind paws [15].

However, this does not rule out the possibility that the increased CXCL12/CXCR4 signaling axis, initiating the pro-regenerative state in cervical DRG neurons induced by a sciatic nerve lesion, contributes to the development of neuropathic pain. It is well known that non-axotomized DRG neurons under the pro-regenerative state display a higher spontaneous activation [51,52,53]. Our published and current results indicate that the regulation of inflammatory mediators, such as the cytokine IL-6 [7,8] or the chemokine CXCL12/CXCR4 signaling axis, plays a role in triggering neuronal regeneration programs even in uninjured DRG neurons. However, these phenotypic changes in uninjured primary sensory neurons, which are associated with the promotion of axon regeneration, are also linked to neuronal hypersensitivity [52,54]. Although these functional alterations may not be overtly manifested in skin hyperalgesia, changes in electrophysiological properties of the remote cervical DRG neurons can create a signaling imbalance in higher afferent structures, ultimately leading to central sensitization and the induction of neuropathic pain [53]. However, this explanation needs further experimental evidence.

In conclusion, the present experimental results confirmed the bilateral upregulation of CXCL12 and CXCR4 proteins and mRNAs in cervical DRG neurons conditioned by unilateral sciatic nerve injury. The CXCR4 signaling, linked to STAT3 activation, is associated with initiation of the neuronal regeneration program and the axon-promoting status in the primary sensory neurons not connected to the injured sciatic nerve. The induced chemokine CXCL12/CXCR4 associated with the axon-promoting status in uninjured cervical DRG neurons is linked with their hyperexcitability and may contribute to the development and maintenance of neuropathic pain. Because DRGs lack a barrier with the liquor in the paraspinal subarachnoid space [55] and haemato-nerve barriers [56], primary sensory neurons can be effectively targeted by systemic or local treatments to alleviate their hypersensitivity. However, the role of hyperexcitable DRG neurons distant from primary nerve injury, along with their potential clinical implications, requires further investigation.

## 4. Materials and Methods

### 4.1. Animals, Surgical Procedure, and Experimental Groups

The experiments were performed in 68 adult male rats (Wistar, 250–280 g, Anlab, Brno, Czech Republic) housed in the animal housing facility of Masaryk University with a 12 h light/dark cycle, at a temperature of 22–24 °C, and maintained under specific pathogen-free conditions. Sterilized standard rodent food and water were provided ad libitum. Animals for surgical procedures were anesthetized with a mixture of ketamine (40 mg/mL) and xylazine (4 mg/mL), administered intraperitoneally at a dose of 0.2 mL/100 g body weight. All surgical procedures were performed under sterile conditions by the same person in accordance with protocols approved by the Animal Care Committee of the Faculty of Medicine, Brno, Czech Republic.

The right sciatic nerve was exposed at the mid-thigh level, ligated with 2 ligatures and cut to achieve complete sciatic nerve transection (CSNT). The proximal nerve stump was fixed into close muscles to protect the distal stump from reinnervation. Muscle and skin closure was performed with 5/0 sutures. In sham-operated rats, the right sciatic nerve was carefully exposed without any lesion.

**Figure 10:**
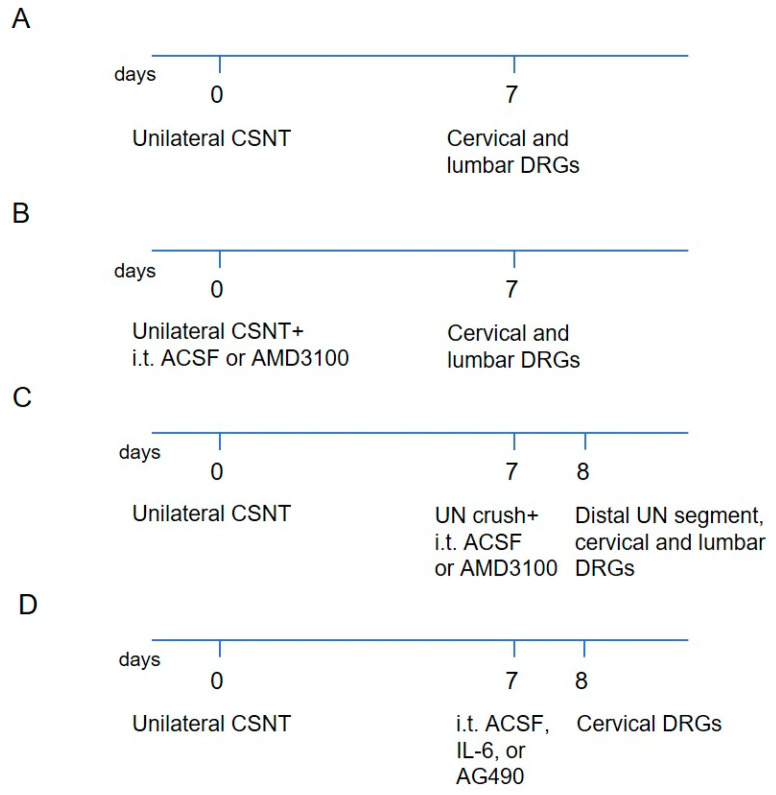
A diagram illustrating the rat experimental groups, timelines, and samples collected from cervical (C7–C8) and lumbar (L4–L5) DRGs, as well as the segments distal to UN crush, for analyses. (**A**) Samples were used for immunohistochemical analysis of CXCL12 and CXCR4 proteins and their mRNAs using RT-PCR. (**B**) Samples were analyzed by immunohistochemistry and western blot for CXCL12 and CXCR4 proteins, as well as immunohistochemical analysis of STAT3. (**C**) Nerve segments distal to the UN crush were used for SCG10 immunohistochemical analysis. (**D**) Cervical DRGs were analyzed for CXCR4 in neuronal nuclei by immunohistochemistry.

Lumbar (L4–L5) and cervical (C7–C8) DRGs were harvested from sham-, and CSNT-operated rats that survived to postoperative day 7 (POD7), as well as from naïve rats, (n = 12 for each group). These samples were used for immunofluorescence detection and RT-PCR analysis of CXCL12 and CXCR4 proteins and mRNAs, respectively. To provide in vivo evidence for the involvement of CXCR4 signaling in the neuronal pro-regeneration program, additional CSNT-operated rats were divided into experimental groups: i. CSNT-operated rats with intrathecal administration of either artificial cerebrospinal fluid (ACSF; [57], or AMD3100 (n = 7 for each group); ii. rats subjected to CSNT on POD7 followed by ulnar nerve (UN) crush and intrathecal administration of ACSF or AMD3100 (n = 4 for each group); iii. CSNT-operated rats with intrathecal administration of IL-6 or AG490, an inhibitor of JAK2 (n = 3 for each group). Additionally, DRGs from 3 naïve rats were used to prepare a primary culture of DRG neurons. The division of rats into experimental groups and the analysis used are summarized in Table S1, while Figure 10 provides an overview of the rat experimental groups, timelines, and samples used for analysis.

### 4.2. Sections, Immunohistochemical Staining, and Semiquantitative Assessment

Rats were deeply anesthetized with a lethal dose of sodium pentobarbital (70 mg/kg body weight, i.p.) and transcardially perfused with 500 mL of heparinized (1000 units/500 mL) phosphate-buffered saline (PBS, pH 7.4), followed by 500 mL of Zamboni’s fixative solution [58]. Lumbar (L4–L5) and cervical (C7–C8) DRGs were removed from both sides and immersed in Zamboni’s fixative overnight at 4 °C. DRGs were divided into the ipsilateral lumbar (L-DRGi), contralateral lumbar (L-DRGc), ipsilateral cervical (C-DRGi), and contralateral cervical (C-DRGc) samples for each group of rats. The samples were then washed in 20% phosphate-buffered sucrose for 12 h, blocked in Tissue-Tek^®^ OCT compound (Miles, Elkhart, IN, USA), and cut to prepare serial longitudinal cryostat sections (12 µm). Sections of ipsilateral and contralateral lumbar and cervical DRGs were mounted on chromium-alum-coated slides and processed for indirect immuno-histochemical staining to detect CXCL12 and CXCR4 immunofluorescence. To compare immunofluorescence intensities, sections of the cervical and lumbar DRGs obtained from naïve and sham controls and CSNT-operated rats were simultaneously immunostained under identical conditions. Briefly, sections were washed with PBS containing 0.05% Tween 20 and 1% BSA for 10 min. They were then treated with 5% normal donkey serum for 30 min and incubated with 25 µL of rabbit polyclonal anti-CXCL12 (1:200, PA1-29029, Thermo Fisher Scientific, Inc., Waltham, MA, USA) or anti-CXCR4 antibody (1:100; LS-C417098, LS Biosciences, Seattle, WA, USA) in a humidity chamber at room temperature (21–23 °C) for 18 h. Immunoreactivity was visualized by treating the sections with TRITC-conjugated and affinity-purified goat anti-rabbit secondary antibody (1:100; Jackson, Ely, UK) for 90 min at room temperature. Control sections were incubated by omitting the primary antibodies or by substituting the primary antibodies with goat IgG isotype. No immunopositive results were obtained.

Immunostained sections were assessed using an epifluorescence microscope (Nikon Eclipse) equipped with a Nikon DS-Ri1 camera (Nikon, Prague, Czech Republic). Images were taken under the same conditions using a stabilized power supply for the lamp housing. Neuronal diameter and CXCL12, CXCR4, and STAT3-IF intensities were measured using the NIS-Elements image analysis system (Nikon, Prague, Czech Republic) as described previously [6,8,18]. Briefly, at least 60 neuronal profiles containing nuclei with distinct nucleoli were measured for each group of animals. The sizes of DRG neurons in sections for immunofluorescence were categorized as small (<25 μm), medium (25–40 μm), and large (>40 μm) according to their diameters calculated from the areas of the neuronal profiles. The immunofluorescence intensities of neuronal cytoplasm and neuronal nuclei were measured separately after subtraction of background and structural detection by a thresholding technique. The binary foregrounds were obtained, monitored at each step of thresholding, and manually edited when necessary. The original color image was converted to gray, overlaid with the binary map, and measured. Results were expressed as mean intensity ± SD. CXCL12 and CXCR4-IF intensities in SGCs were described only in comparison to those in neurons.

### 4.3. Real-Time PCR

We analyzed the levels of CXCL12 and CXCR4 mRNAs in DRGs using real-time PCR (RT-PCR). Whole DRGs were harvested under aseptic conditions from both lumbar (L4–L5) and cervical (C7–C8) segments of naïve and sham-, SNC- and CSNT-operated rats (n = 3 for each group in three independent experiments). The samples, collected as ipsilateral and contralateral, were subsequently stored in RNA (Thermo Fisher Scientific, Inc., Waltham, MA, USA) at 4 °C. First-strand synthesis was performed using the TaqMan^®^ High-Capacity RNA-to-cDNA Kit, and the quality and concentration were assessed by optical density using NanoDrop. PCR amplification was performed in triplicate for each sample using the ABI Prism 7300, TaqMan^®^ Gene Expression Master Mix, and TaqMan^®^ Gene Expression Assay Probes FAM™ (Thermo Fisher Scientific, Inc., Waltham, MA, USA) for the target gene CXCL12 (Assay ID-Rn00573260_m1) or CXCR4 (Assay ID-Rn00573522_s1). The determination was performed with reference to the reporter gene encoding GAPDH (assay ID-Rn01775763_g1) Endogenous Control (VIC^®^). The polymerase activation step at 95 °C for 15 min was followed by 40 cycles of 15 s at 95 °C and 60 s at 60 °C. The validity of the results was confirmed by performing appropriate negative controls, including water instead of cDNA for PCR amplification and omitting reverse transcriptase for cDNA synthesis. Specific mRNA levels were calculated after normalization to actin mRNA in each sample. Relative expression was determined using the comparative Ct model (ΔΔCt) with actin as the housekeeping gene. Data are presented as relative mRNA units compared with control values, expressed as fold over the naïve value.

### 4.4. Analysis of CXCR4 in Relation to the Pro-Regenerative State of Cervical DRG Neurons Induced by a Sciatic Nerve Lesion

To demonstrate the effect of sciatic nerve lesion on the CXCR4 signaling axis associated with the pro-regenerative state of cervical DRG neurons, rats in the first experiment were subjected to CSNT (n = 14) and divided into two groups. Rats in the control group (n = 7) were administered with ACSF, while the second group of rats (n = 7) received AMD3100. Ten µL of ACSF or freshly prepared AMD3100 (Sigma, Saint Louis, MO, USA) in ACSF (5 mg/kg), followed by an additional 10 µL of ACSF, was injected into the subarachnoid space of the cisterna magna using a microsyringe. The dose of AMD3100 was determined in previous studies [15,44]. All rats were allowed to survive until POD7.

#### 4.4.1. Immunofluorescence Staining

Three rats from each group (ACSF and AMD3100) were transcardially perfused with Zamboni’s fixative solution. Cervical DRGs (C7–C8) and lumbar DRGs (L4–L5) were removed from both sides, washed in 20% phosphate-buffered sucrose, and longitudinal cryostat sections were cut. The sections were processed for CXCL12 and CXCR4 immunostaining as described above.

#### 4.4.2. Western Blot Analysis

For Western blot analysis, naïve rats (n = 4), and rats subjected to CSNT on POD7 and treated with intrathecal administration of ACSF (n = 4) and AMD3100 (n = 4) were deeply anesthetized with a lethal dose of sodium pentobarbital (70 mg/kg body weight, i.p.). Fresh cervical (C7–C8) and lumbar (L4–L5) DRGs were removed bilaterally under aseptic conditions, washed in protease and phosphatase inhibitor cocktails (both from LaRoche, Basel, Switzerland), snap frozen in liquid nitrogen, and stored at −80 °C. DRG samples were homogenized in TRIS-buffered saline (pH 7.2) with 0.1% Triton X-100 and cocktail of protease and phosphatase inhibitors (LaRoche, Basel, Switzerland), then centrifuged at 10,000× *g* for 5 min at 4 °C. Proteins were separated by SDS-polyacrylamide gel electrophoresis and transferred to nitrocellulose membranes using electroblotting (Bio-Rad, Hercules, CA, USA). After blocking with 5% bovine serum albumin (BSA) in TRIS-buffered saline (pH 7.2) for 2 h, the membranes were incubated overnight with rabbit polyclonal anti-CXCL12 (1:100; Thermo Fisher Sci., Waltham, MA, USA) or anti-CXCR4 (1:100; LS Biosciences Seattle, WA, USA) antibodies. Blots were washed in TRIS-buffered saline (pH 7.2) and incubated with peroxidase-conjugated anti-rabbit IgG (1:1000; Sigma, Saint Louis, MO, USA) for 1 h at room temperature. Protein bands were visualized using the ECL detection kit (Amersham, Piscataway, NJ, USA) on the chemiluminometer reader LAS-3000 (Fuji Photo Film, Tokyo, Japan) and analyzed using its densitometry image software. Protein levels were normalized to β-actin, which was used as a housekeeping protein, and then further normalized to the value of naïve DRGs, which was set to one.

#### 4.4.3. In Vivo Assay of Axon Regeneration

For in vivo evidence that sciatic nerve lesion triggers the CXCR4 signaling axis associated with the pro-regenerative state of cervical DRG neurons, we use our experimental model with a prior sciatic nerve lesion for 7 days followed by the ulnar nerve (UN) crush [8]. Briefly, rats with prior CSNT (n = 8) on POD7 were re-operated. A short segment of the right UN was exposed and crushed using a clamp with a defined force of 1.9 N for 2 periods of 1 min each [59] under a stereoscopic microscope. The distal margin of the crush injury was marked with a 10-0 epineural suture. The skin wound was closed with 5/0 sutures, and the rats were randomly divided into two groups: a control group (n = 4) receiving intrathecal administration of ACSF, and a group receiving intrathecal administration of AMD3100 (n = 4) as described above. Rats were allowed to survive for 1 day, deeply anesthetized, and transcardially perfused with Zamboni’s fixative solution. Nerve segments distal to the UN crush were removed, washed in 10% phosphate-buffered sucrose, and cryostat sectioned longitudinally (10 µm thick). Axon regeneration was assessed on sections immunostained with a rabbit polyclonal antibody against SCG-10 (1:1000; LS Biosciences, Seattle, WA, USA) in a humid chamber at room temperature (21–23 °C) for 12 h. The immunoreaction was visualized by treatment with FITC-conjugated and affinity-purified donkey anti-rabbit secondary antibody (1:100; Millipore, Burlington, MA, USA) for 90 min at room temperature. The fluorescence intensity of SCG-10 was analyzed along the length of the nerve distal to the crush site [60]. The length of SCG-10-immunopositive axons was measured in every third section by a person blinded to the experimental conditions using the NIS-Elements image analysis system (Nikon, Prague, Czech Republic), and analyzed as previously described [7,8].

#### 4.4.4. In Vitro Assessment of Axonal Outgrowth in Cervical DRG Neurons Associated with the CXCR4 Signaling Axis

A modified protocol [61] was used to prepare an in vitro culture of dissociated cervical DRG neurons. Three rats were deeply anesthetized by intraperitoneal administration of sodium pentobarbital (70 mg/kg) and subsequently euthanized by decapitation. Cervical DRGs (C7–C8) were harvested bilaterally under aseptic conditions after laminectomy. DRGs were collected in ice-cold Ca^2+^/Mg^2+^-free Hank’s buffered saline (CMF-HBSS, Sigma-Aldrich, Saint Louis, MO, USA). After removal the connective tissue, DRGs were dissociated. This involved treatment with a medium containing 0.1% collagenase type I (5000 U/mL) for 90 min, followed by 0.25% trypsin/EDTA at 37 °C for 25 min. The resulting suspension of DRG neurons was prepared by triturating through glass pipette tips and washed twice with Dulbecco’s Modified Eagle’s Medium/Nutrient F-12 Ham (DMEM/F12) supplemented with 10% fetal bovine serum (FBS; all from Sigma Aldrich, Saint Louis, MO, USA). After centrifugation at 1500 rpm for 5 min at 4 °C, the cells were resuspended and placed into a culture medium of DMEM/F12 supplemented with 2 mM glutamine, 100 U/mL penicillin and 100 μg/mL streptomycin (all from Sigma Aldrich, Saint Louis, MO, USA), as well as N2 and B27 (Thermo Fisher Sci., Waltham, MA, USA; diluted according to the manufacturer’s instructions). The DRG neurons were then reseeded at a density of 200 cells on glass coverslips previously coated with Geltrex^®^ (Thermo Fisher Sci., Waltham, MA, USA). They were then incubated for 2 days at 37 °C in a humidified atmosphere containing 5% CO_2_.

For treatment, DRG neurons were cultured in a medium containing 10 ng/mL CXCL12 (PeproTech, Rocky Hill, NJ, USA) or CXCL12 (10 ng/mL) applied with AMD3100 (100 ng/mL, Sigma-Aldrich, Saint Louis, MO, USA), both for 6 h. Control neurons without any treatment were cultured in parallel and processed at the same time points as the treated neurons. Coverslips containing neurons were washed in PBS, fixed in Zamboni solution, immunostained with mouse anti-β-tubulin III primary antibody (Sigma, Saint Louis, MO, USA; 1:500), and analyzed in at least 20 randomly selected nucleate neurons per experimental group [8]. The mean number of neurites per neuron and the total neurite length per neuron were calculated from triplicate experiments, and data were presented as mean ± SD.

#### 4.4.5. Relationship Between the CXCR4 Signaling Axis and the Activation of STAT3 in DRG Neurons of In Vivo Experimental Rats

To investigate the role of the CXCR4 signaling axis in the activation of STAT3, serving as a marker for the initiation of a pro-regenerative state induced by sciatic nerve injury in cervical DRG neurons, we performed additional experiments and analyses. First, we used longitudinal sections through cervical DRGs of rats subjected to CSNT on POD7 followed by intrathecal administration of either ACSF or AMD3100 (see above). Sections were immunostained under identical conditions to detect and quantify nuclear STAT3 phosphorylated at the tyrosine-705 (Y705) position [18].

In further experiments to investigate the relationship between the CXCR4 signaling axis and STAT3 activation, intrathecal administration of IL-6, as an activator, or AG490 as an inhibitor, of STAT3 activation, was performed in rats subjected to CSNT on POD7. Recombinant rat IL-6 protein (R&D Systems, Minneapolis, MN, USA) was dissolved in ACSF at a concentration of 20 ng/10 µL. Additionally, AG490 (Sigma, Saint Louis, MO, USA), an inhibitor of JAK2, was prepared at a concentration of 5µM. A solution of IL-6 (10 µL), AG490 (10 µL), or ACSF (10 µL), along with an additional 10 µL ACSF, was injected into the subarachnoid space of the cisterna magna using a microsyringe (n = 3 for each group). Animals were allowed to survive for 1 day, after which, cervical DRGs (C7–C8) were harvested after pericardial perfusion with Zamboni fixative solution. Longitudinal sections of DRGs from all experimental groups were immunostained under identical conditions for the detection and quantification of CXCR4, as previously described. The intensities of CXCR4-IF were measured in the neuronal nuclei using the NIS-Elements image analysis system (Nikon, Prague, Czech Republic) according to our published protocol [18].

#### 4.4.6. Double Immunostaining Distal to the Ulnar Nerve Crush

The cellular distribution of CXCR4 and CXCL12 was detected in longitudinal sections of the nerve segment distal to the UN crush using double immunostaining. The sections were incubated with mouse monoclonal anti-GAP43 (1:500) and rabbit anti-CXCR4 (1:100) or rabbit anti-CXCL12 (1:100) primary antibodies. After thorough washing, a mixture (1:1) of affinity-purified FITC-conjugated goat anti-mouse and TRITC-conjugated goat anti-rabbit secondary antibodies (Millipor, Billerica, MA, USA) was applied at a final dilution of 1:100 for 90 min at room temperature to visualize primary antibody binding.

To determine the distribution of CXCL12 in non-neuronal cell types near growth cones, sections were incubated with rabbit anti-CXCL12 (1:100) and chicken anti-GFAP (1:500) primary antibodies, followed by affinity-purified TRITC-conjugated goat anti-rabbit and affinity-purified FITC-conjugated goat anti-chicken secondary antibodies. Control sections were incubated without primary antibodies or with reversed combination of primary and secondary antibodies. No immunopositive results were obtained in the control sections.

#### 4.4.7. Statistical Analyses

The Mann–Whitney U test was used to assess statistical differences in immunofluorescence intensities, Western blot, and RT-PCR data among values obtained from naïve, sham-operated, and sciatic nerve lesion-operated rats. Statistical significance was considered at *p*-value < 0.05 (*), *p*-value < 0.005 (**), and *p*-value < 0.001 (***). To compare the mean number of neurites per neuron and total neurite length per neuron between control dorsal root ganglia (DRG) neurons and those treated with CXCL12, we utilized one-way ANOVA followed by Tukey’s post hoc test. Similarly, comparisons were made between CXCL12-treated and CXCL12/AMD3100-treated neurons. Statistical analyses were performed using STATISTICA 12 software (StatSoft, Inc., Tulsa, OK, USA).

## Data Availability

The original contributions presented in the study are included in the article; further inquiries can be directed to the corresponding author.

## Supplementary Materials

The supporting information can be downloaded at: https://www.mdpi.com/article/10.3390/ijms26010193/s1.
