# Supplementary material for: The Intrinsic Neuronal Activation of the CXCR4 Signaling Axis Is Associated with a Pro-Regenerative State in Cervical Primary Sensory Neurons Conditioned by a Sciatic Nerve Lesion
Source: Int J Mol Sci. 2024 Dec 29;26(1):193. doi: 10.3390/ijms26010193 (PMC11720091; doi:10.3390/ijms26010193)
Supplement: Supplementary file 1 [file ijms-26-00193-s001.zip › Table S1.pdf]

Table S1. Number of rats per experimental group and analysis. Complete sciatic nerve transection for 7 post-operation days (CSNT POD7); ulnar nerve (UN) crush; intrathecal application (i.t. appl); immunohistochemical staining (IHC); real time PCR (RT-PCR); western blot (WB).

**Experimental group of rats**	**IHC**	**RT-PCR**	**WB**	**In vivo**
Naive	3	9	4	-
Sham operation	3	9	-	-
CSNT POD7	3	9	-	-
CSNT POD7 + i.t. appl. ACSF	3	-	4	-
CSNT POD7 + i.t. appl. AMD3100	3	-	4	-
CSNT POD7 + UN crush + i.t. appl. ACSF	-	-	-	4
CSNT POD7 + UN crush + i.t. appl. AMD3100	-	-	-	4
CSNT POD7 + i.t. appl. IL-6	3	-	-	-
CSNT POD7 + i.t. appl. AG490	3	-	-	-
